# Tissue-based detection of macrophage-associated YAP1-positive material in human acute liver injury: an exploratory immunopathological biopsy series

**DOI:** 10.1186/s13000-026-01805-8

**Published:** 2026-07-16

**Authors:** Hiroteru Kamimura, Kenya Kamimura, Shuji Terai

**Affiliations:** Division of Gastroenterology and Hepatology, Niigata University Graduate School of Medicine, Dentistry and Health Sciences (Medicine), Niigata University, 1-757 Asahimachi-dori, Chuo-ku, Niigata, 951-8510 Japan

**Keywords:** Acute liver injury, CD68, Ductular reaction, Immunofluorescence, Kupffer cell, Liver biopsy, Yes-associated protein 1

## Abstract

**Background:**

Yes-associated protein 1 (YAP1), a downstream effector of the Hippo pathway, is implicated in hepatocellular stress, ductular reaction, and tissue repair. Experimental studies suggest that injured YAP1-activated hepatocytes can be cleared by Kupffer-cell phagocytosis, but corresponding human biopsy evidence remains limited.

**Methods:**

We retrospectively analyzed seven adults who underwent clinically indicated liver biopsy within 10 days of initial presentation or referral admission for acute liver injury. Liver biopsy sections were assessed by hematoxylin and eosin staining, periodic acid-Schiff-diastase staining, YAP1 immunohistochemistry, and double immunofluorescence for YAP1 and CD68. Macrophage-associated YAP1-positive structures were operationally defined as YAP1-positive signals located within or immediately adjacent to CD68-positive macrophages in portal or periportal areas. The definition was intended to capture spatial association and was not intended to prove intracellular phagocytosis, molecular co-expression, or endogenous YAP1 expression by macrophages. Associations with peak Model for End-Stage Liver Disease (MELD) score and days to alanine aminotransferase (ALT) normalization were evaluated as exploratory analyses.

**Results:**

The median age was 52 years, and etiologies included autoimmune hepatitis, primary biliary cholangitis-autoimmune hepatitis overlap, drug-induced liver injury, acute hepatitis B, and idiopathic acute liver injury. Liver biopsy was performed at a median of 5 days (range, 2–10 days) after initial presentation or referral admission. A median of nine portal tracts and five macrophage-associated YAP1-positive structures were evaluated per biopsy, yielding a median YAP1/CD68-positive portal-tract index of 58.3%. ALT normalized after a median of 26 days. The index showed a nominal inverse association with days to ALT normalization (Spearman rho = -0.86, *p* = 0.014; Pearson r = -0.79, *p* = 0.034), whereas MELD score was not significantly associated with the index or recovery time. Leave-one-out analyses retained the inverse direction but showed unstable significance because of the very small sample size.

**Conclusions:**

In this small exploratory biopsy series, YAP1-positive material was observed in spatial association with CD68-positive macrophages in early human acute liver injury. The YAP1/CD68-positive portal-tract index showed a hypothesis-generating relationship with biochemical recovery but should not be interpreted as a validated prognostic marker or proof of macrophage-mediated phagocytosis. Larger, etiology-specific studies with standardized biopsy timing, lineage markers, multiplex immunofluorescence, high-resolution imaging, and digital pathology are warranted.

## Background

The liver can recover rapidly from acute injury, but biopsy-based features that distinguish effective tissue repair from delayed biochemical recovery remain incompletely defined. The Hippo pathway effector YAP1 regulates organ growth, cell fate, and tissue remodeling, and its activation has been described in hepatocytes, biliary/progenitor-like compartments, and regenerative liver lesions [1–3]. In the setting of injury, YAP1 should therefore be interpreted not merely as a proliferation marker, but as a context-dependent signal linked to cellular stress, plasticity, and repair.

An important experimental observation is that injured YAP1-activated hepatocytes can migrate into sinusoids, undergo apoptosis, and be engulfed by Kupffer cells [4]. This experimental cell-removal mechanism provides a biological rationale for examining whether YAP1-positive cellular material is spatially associated with macrophages in human liver biopsy tissue. However, most human studies have focused on YAP1 expression in epithelial or progenitor-like compartments rather than on the spatial relationship between YAP1-positive material and macrophages.

Ductular reaction is a common histological response to severe hepatobiliary injury. It comprises reactive ductular cells, progenitor-like cells, and hepatocytes undergoing phenotypic plasticity, with accompanying inflammatory and stromal remodeling [5–7]. Depending on the microenvironment, ductular reaction may facilitate epithelial repair, but persistent or excessive ductular reaction is also linked to inflammation, fibrogenesis, and disease progression [6–8]. Macrophage-epithelial crosstalk is thus likely to be central to the balance between resolution and progression.

We hypothesized that YAP1-positive material spatially associated with CD68-positive macrophages in portal or periportal areas may provide an exploratory tissue-based observation of the local injury-repair microenvironment in acute liver injury. This biopsy series examined whether such a feature is detectable in early human acute liver injury and whether it relates to the time required for ALT normalization.

## Methods

### Study design and patients

This retrospective exploratory biopsy series included seven adult patients who underwent clinically indicated liver biopsy within 10 days of initial presentation or referral admission for acute liver injury at Niigata University. Because exact symptom-onset dates were not uniformly reliable in the retrospective records, biopsy timing was defined as the interval from initial presentation or referral admission to liver biopsy. Etiologies were determined from clinical presentation, serological testing, histological findings, medication history, and treatment response. The cohort included drug-induced liver injury, autoimmune hepatitis, primary biliary cholangitis-autoimmune hepatitis overlap, acute hepatitis B, and idiopathic acute liver injury. Peak MELD score during the acute episode was recorded as a systemic severity marker. Biochemical recovery was defined as normalization of serum ALT according to the institutional reference range, and the number of days from clinical onset or initial recognition of acute liver injury to ALT normalization was used for analysis. In the two patients with primary biliary cholangitis-autoimmune hepatitis overlap, treatment was selected according to the dominant clinical phenotype and disease activity; both recovered without corticosteroid therapy.

### Histology and immunofluorescence

Formalin-fixed, paraffin-embedded liver biopsy sections were evaluated by hematoxylin and eosin staining to assess hepatocellular injury, inflammatory infiltration, and ductular reaction. Periodic acid-Schiff-diastase staining was used to identify macrophages with cytoplasmic cellular material in portal areas. For immunofluorescence analysis, YAP1 was detected using anti-YAP1 antibody [EP1674Y] (Abcam, ab52771; recombinant rabbit monoclonal), and macrophages were labeled using anti-CD68 antibody [KP1] (Agilent/Dako, M0718; mouse monoclonal). After primary antibody incubation, sections were incubated with goat anti-rabbit IgG conjugated with Cy3 for YAP1 and goat anti-mouse IgG conjugated with FITC for CD68. Nuclei were counterstained with DAPI.

For each biopsy, all evaluable portal tracts were assessed. Macrophage-associated YAP1-positive structures were operationally defined as YAP1-positive signals located within CD68-positive macrophages or immediately adjacent to them in portal or periportal areas on merged immunofluorescence images. This definition was intended to capture spatial association between YAP1-positive material and CD68-positive macrophages. It was not intended to demonstrate intracellular phagocytosis, molecular co-expression within a single cell, or endogenous YAP1 expression by macrophages. The number of evaluable portal tracts and the number of macrophage-associated YAP1-positive structures were counted across all evaluable portal tracts using the same operational definition throughout. Counting was performed by the primary investigator, and the images and counts were reviewed with a second author for consistency; discrepant or ambiguous structures were resolved by consensus review. Formal blinded interobserver reproducibility testing was not performed. A YAP1/CD68-positive portal-tract index was calculated as the number of macrophage-associated YAP1-positive structures divided by the number of portal tracts examined, multiplied by 100.

### Statistical analysis

Continuous variables are presented as medians and ranges. Because of the very small sample size and exploratory nature of the study, Spearman correlation was used as the primary exploratory analysis for associations among the YAP1/CD68-positive portal-tract index, peak MELD score, and days to ALT normalization. Pearson correlation coefficients with 95% confidence intervals calculated using Fisher’s z transformation were also reported as secondary sensitivity analyses. No adjustment for multiple testing was performed; therefore, all *p* values should be interpreted as nominal and hypothesis-generating. To assess whether the direction of the association between the YAP1/CD68-positive portal-tract index and days to ALT normalization depended on a single observation, leave-one-out sensitivity analyses were performed by repeating the correlation analysis after excluding each patient one at a time. Analyses were performed using GraphPad Prism version 9.0 (GraphPad Software, San Diego, CA, USA) and R version 4.4.3.

## Results

### Clinical characteristics

The clinical characteristics are summarized in Table 1. The median age was 52 years (range, 41–61 years), and three patients were male. Etiologies included autoimmune hepatitis in two patients, primary biliary cholangitis-autoimmune hepatitis overlap in two patients, and drug-induced liver injury, acute hepatitis B, and idiopathic acute liver injury in one patient each. Liver biopsy was performed within 10 days of initial presentation or referral admission in all cases, with a median interval of 5 days (range, 2–10 days). All patients recovered after etiology-directed management or observation. The median peak MELD score was 20 (range, 13–23). ALT normalized after a median of 26 days (range, 11–78 days).

**Table 1:** Clinical characteristics, biopsy timing, and portal-tract YAP1/CD68 positivity in seven patients with acute liver injury

Case	Age/Sex	Etiology	Peak MELD	Biopsy timing, days^*^	Portal tracts, n	YAP1 + structures, n	YAP1/CD68 index, %	Days to ALT normalization; management/outcome
1	61/M	DILI	14	2	8	8	100.0	11; medication withdrawal; recovered
2	55/F	AIH	22	3	12	7	58.3	20; prednisolone; recovered
3	52/F	AIH	20	4	11	7	63.6	26; prednisolone; recovered
4	48/F	PBC-AIH overlap	13	5	8	5	62.5	22; ursodeoxycholic acid; recovered
5	50/F	PBC-AIH overlap	15	7	7	3	42.9	60; ursodeoxycholic acid; recovered
6	52/M	Acute hepatitis B	23	10	10	4	40.0	78; nucleos(t)ide analogue; recovered
7	41/M	Idiopathic acute liver injury	23	6	9	5	55.6	31; observation; recovered

*Abbreviations*: *AIH* autoimmune hepatitis, *DILI* drug-induced liver injury, *F* female, *M* male, *MELD* Model for End-Stage Liver Disease, *PBC* primary biliary cholangitis, *YAP1* Yes-associated protein 1

^*^Biopsy timing was defined as the interval from initial presentation or referral admission to liver biopsy

### Histological and immunopathological findings

**Fig. 1:**
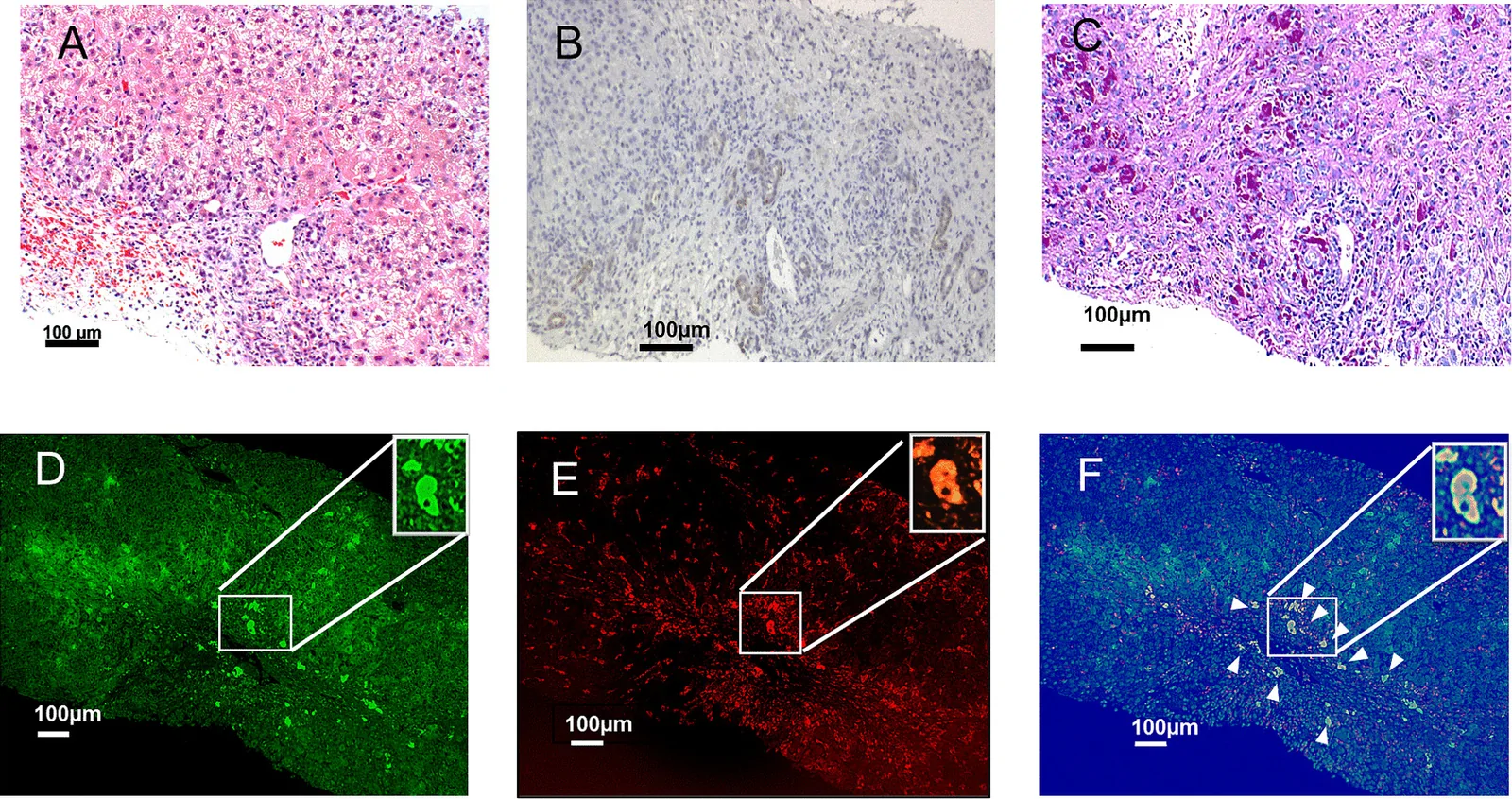
Histological and immunofluorescence findings in acute liver injury. **A** Hematoxylin and eosin staining showing periportal hepatocellular injury with inflammatory cell infiltration. **B** YAP1 immunohistochemistry showing YAP1 expression in ductular reaction and periportal hepatocytes. **C** Periodic acid-Schiff-diastase staining highlighting macrophages with cytoplasmic cellular material in the portal area. **D**-**F** Double immunofluorescence staining for CD68 and YAP1. CD68-positive macrophages are shown in green (**D**), YAP1-positive signals in red (**E**), and the merged image with DAPI nuclear counterstaining in blue (**F**). YAP1-positive material was observed in spatial association with CD68-positive macrophages. Arrowheads indicate representative macrophage-associated YAP1-positive structures, and the inset shows a high-magnification view of the boxed area. Scale bars, 100 µm

A median of nine portal tracts per biopsy (range, 7–12) and five macrophage-associated YAP1-positive structures (range, 3–8) were evaluated, yielding a median YAP1/CD68-positive portal-tract index of 58.3% (range, 40.0–100.0%). Biopsy specimens obtained during the acute phase showed periportal hepatocellular injury with inflammatory infiltration and ductular reaction. YAP1 immunohistochemistry demonstrated YAP1 expression in ductular reaction and periportal hepatocytes. Double immunofluorescence showed CD68-positive macrophages in portal/periportal areas and YAP1-positive material in close spatial association with these macrophages (Fig. 1A-F).

### Exploratory correlation with biochemical recovery

**Fig. 2:**
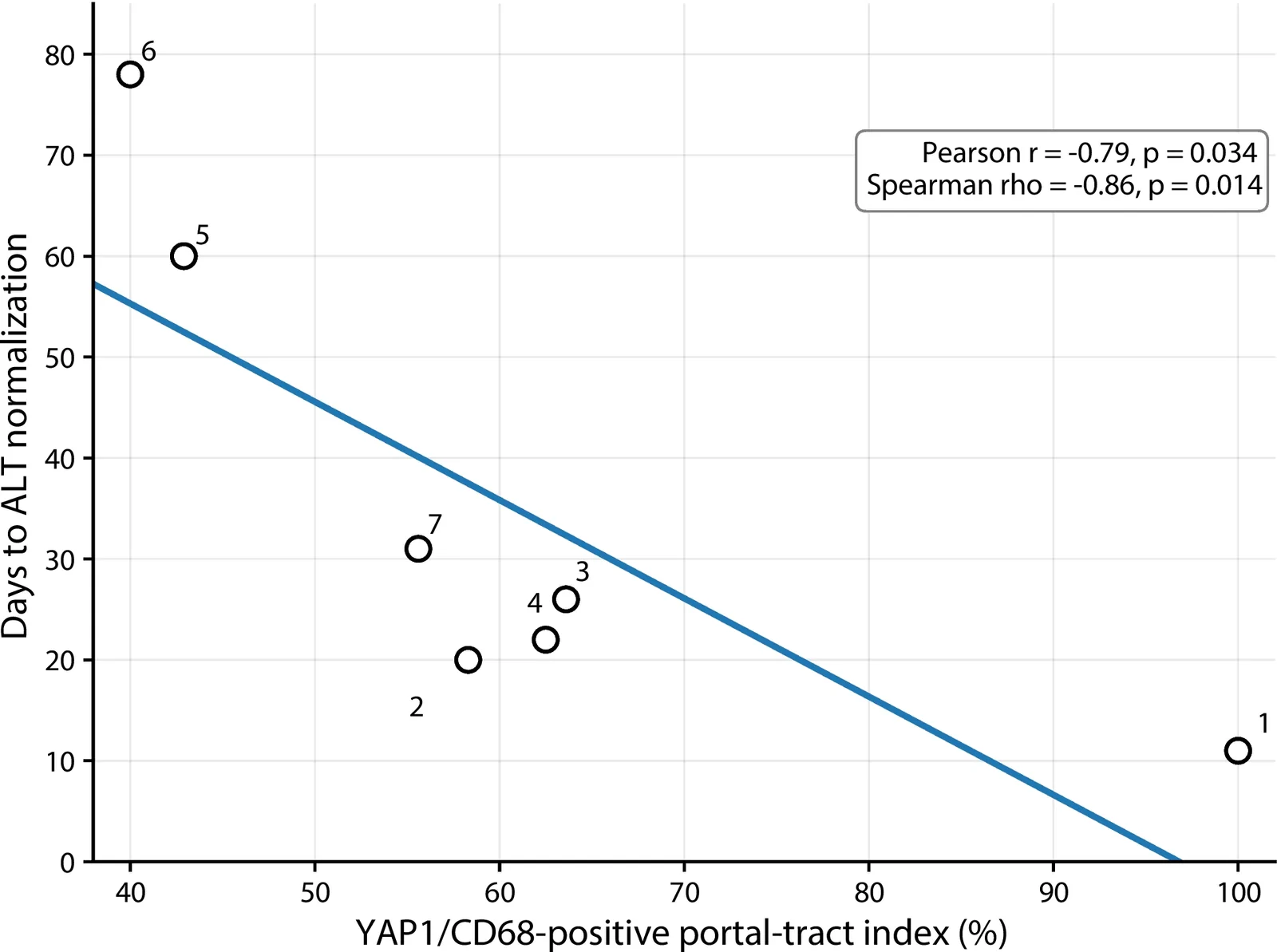
Relationship between the YAP1/CD68-positive portal-tract index and biochemical recovery. Scatter plot showing the relationship between the YAP1/CD68-positive portal-tract index and days to alanine aminotransferase normalization. The index showed a nominal inverse association with days to alanine aminotransferase normalization. This analysis was exploratory, and *p* values should be interpreted as nominal because of the very small sample size and absence of adjustment for multiple testing

Correlation analyses are summarized in Table 2. The YAP1/CD68-positive portal-tract index showed a nominal inverse association with days to ALT normalization (Spearman rho = −0.86, *p* = 0.014; Pearson r = −0.79, 95% CI −0.97 to −0.09, *p* = 0.034) (Fig. 2). In leave-one-out sensitivity analyses, the inverse direction of the association was retained after exclusion of each individual patient (Spearman rho range, −0.94 to −0.77; Pearson r range, −0.95 to −0.75), although nominal *p* values were unstable because of the very small sample size. In contrast, peak MELD score was not significantly associated with the YAP1/CD68-positive portal-tract index (Spearman rho = −0.61, *p* = 0.144; Pearson r = −0.47, *p* = 0.29) or days to ALT normalization (Spearman rho =0.54 , *p* =0.210 ; Pearson r = 0.31, *p* = 0.50). These analyses were considered descriptive and hypothesis-generating.

**Table 2:** Exploratory correlation analysis among disease severity, portal-tract YAP1/CD68 positivity, and ALT normalization

Variable 1	Variable 2	Spearman rho	*p* value	Pearson r (95% CI)	*p* value	Interpretation
MELD score	YAP1/CD68-positive portal-tract index (%)	-0.61	0.144	−0.47 (−0.90 to 0.44)	0.29	No nominal association
MELD score	Days to ALT normalization	0.54	0.210	0.31 (−0.58 to 0.86)	0.50	No nominal association
YAP1/CD68-positive portal-tract index (%)	Days to ALT normalization	−0.86	0.014	−0.79 (−0.97 to −0.09)	0.034	Nominal inverse association

No adjustment for multiple testing was performed; therefore, all *p* values should be interpreted as nominal and hypothesis-generating. Leave-one-out sensitivity analysis for the YAP1/CD68-positive portal-tract index and days to ALT normalization retained the inverse direction after exclusion of each individual patient (Spearman rho range, −0.94 to −0.77; Pearson r range, −0.95 to −0.75)

*Abbreviations*: *ALT* alanine aminotransferase, *CI* confidence interval, *MELD* Model for End-Stage Liver Disease, *YAP1* Yes-associated protein 1

## Discussion

This exploratory human biopsy series suggests that YAP1-positive material can be detected in spatial association with CD68-positive macrophages in portal/periportal areas during early acute liver injury. The principal observation is not YAP1 expression itself, which has already been linked to hepatocellular stress, cell fate regulation, and ductular reaction, but the spatial relationship between YAP1-positive material and CD68-positive macrophages. This finding should be viewed as an exploratory tissue-based observation of the local injury-repair microenvironment rather than as evidence of a validated prognostic marker or proven macrophage-mediated phagocytosis.

The observation is biologically plausible but remains inferential. Yimlamai et al. showed that Hippo pathway activity influences liver cell fate, including hepatocyte and biliary/progenitor-like phenotypes [3]. Miyamura et al. subsequently demonstrated that YAP1 activation in injured mouse hepatocytes promotes selective elimination of these cells, which migrate into sinusoids, undergo apoptosis, and are engulfed by Kupffer cells [4]. These experimental findings provide a mechanistic framework for examining YAP1-positive material in spatial association with CD68-positive macrophages in human biopsy tissue. However, the cellular origin of the YAP1-positive material in the present human series remains uncertain. Importantly, acetaminophen-induced liver injury models also suggest that YAP1-related signaling is context dependent: hepatocyte-specific YAP deletion improved recovery in one model, whereas an incremental-dose model identified coordinated pro-regenerative signaling during recovery [9, 10]. Thus, YAP1-positive signals in acute liver injury may mark a dynamic stress-repair compartment rather than uniformly beneficial proliferation.

Ductular reaction and macrophage-epithelial crosstalk provide an additional interpretive framework. The ductular reaction has long been recognized as a structured histological response involving the finer branches of the biliary tree and adjacent parenchymal compartments [5]. Subsequent work has emphasized that ductular reaction may support epithelial repair but also contribute to inflammation, fibrosis, and progression when persistent [6, 7]. Terada et al. reported that Kupffer cells can induce Notch-mediated hepatocyte conversion in an experimental liver disease model [11], and Yang et al. showed that targeting the macrophage Notch1-YAP circuit reprograms macrophage polarization and alleviates acute liver injury in mice [12]. Together, these studies support the hypothesis that portal macrophages, ductular reaction, and YAP1-positive cellular material may be spatially linked in the early injury-repair microenvironment. This hypothesis requires validation in disease-specific human cohorts.

From a diagnostic pathology perspective, the present data suggest that macrophage-associated YAP1-positive material may be an exploratory tissue-based feature that complements conventional systemic severity indices. MELD score reflects systemic dysfunction, whereas a biopsy-based YAP1/CD68-positive portal-tract index may capture a local microenvironmental feature within the injured portal/periportal compartment. However, the present findings should not be used for clinical decision-making and should not be interpreted as establishing a diagnostic or prognostic biomarker. The nominal correlation with ALT normalization should instead be used to formulate testable hypotheses for larger digital pathology studies of acute liver injury.

This study has important limitations. First, the sample size was extremely small, and the cohort included etiologically heterogeneous forms of acute liver injury, including autoimmune hepatitis, primary biliary cholangitis-autoimmune hepatitis overlap, drug-induced liver injury, acute hepatitis B, and idiopathic acute liver injury. These disorders differ in pathogenesis, inflammatory pathways, treatment approaches, and recovery kinetics; therefore, pooling them may obscure disease-specific biology and may produce unstable estimates. Second, liver biopsy timing was clinically determined, and serial biopsy material was not available to assess temporal changes. Although biopsy timing from initial presentation or referral admission was added, exact symptom-onset timing was not uniformly reliable in the retrospective records. Third, conventional immunofluorescence without confocal z-stack imaging cannot definitively distinguish true intracellular engulfment from close membrane apposition. Therefore, the present findings should be interpreted strictly as spatial association between YAP1-positive material and CD68-positive macrophages, not as proof of intracellular phagocytosis or macrophage YAP1 expression. Fourth, the cellular origin of the YAP1-positive material remains uncertain because lineage markers such as CK19, SOX9, EpCAM, hepatocyte markers, apoptosis markers, and cell-type-specific quantification of nuclear versus cytoplasmic YAP1 were not examined. Fifth, no normal liver controls or comparator inflammatory liver disease groups were included, so the specificity of this finding for acute liver injury cannot be determined. Finally, the YAP1/CD68-positive portal-tract index was investigator-defined. Formal blinded interobserver reproducibility testing and automated digital quantification were not performed; therefore, this metric requires independent validation before diagnostic or prognostic application.

Future studies should validate this observation in larger, etiology-specific cohorts with standardized biopsy timing. Multiplex immunofluorescence incorporating epithelial markers such as cytokeratin 19, SOX9, and EpCAM, hepatocyte markers, apoptosis markers such as cleaved caspase-3 or CK18-M30, and macrophage markers such as CD163, CD206, and inflammatory macrophage markers would clarify the cellular origin and biological meaning of macrophage-associated YAP1-positive material. Confocal z-stack imaging, digital pathology, and spatial transcriptomic approaches may further define the cellular neighborhoods surrounding YAP1-positive cells and CD68-positive macrophages.

## Conclusions

In this small exploratory biopsy series, YAP1-positive material was observed in spatial association with CD68-positive macrophages in portal/periportal areas of early human acute liver injury. The YAP1/CD68-positive portal-tract index showed a hypothesis-generating inverse relationship with time to ALT normalization, but this finding should not be interpreted as evidence of a validated prognostic marker or proven macrophage-mediated phagocytosis. Larger, etiology-specific studies with standardized biopsy timing, lineage markers, multiplex immunofluorescence, high-resolution imaging, and digital pathology are required.

## Data Availability

The individual-level data generated and analysed during the current study are not publicly available because of patient privacy considerations but may be available from the corresponding author on reasonable request and subject to institutional ethical approval.

## Abbreviations

AIH: Autoimmune hepatitis
ALT: Alanine aminotransferase
CD68: Cluster of differentiation 68
DAPI: 4′,6-Diamidino-2-phenylindole
DILI: Drug-induced liver injury
DR: Ductular reaction
FITC: Fluorescein isothiocyanate
MELD: Model for End-Stage Liver Disease
PBC: Primary biliary cholangitis
YAP1: Yes-associated protein 1
